# Intranasal glial heterotopia in a male infant: A case report

**DOI:** 10.1097/MD.0000000000021200

**Published:** 2020-07-17

**Authors:** Wei Zhang, Lixing Tang, Pengpeng Wang, Wentong Ge, Xin Ni

**Affiliations:** Department of Otorhinolaryngology, Head and Neck Surgery, Beijing Children's Hospital, Capital Medical University, National Center for Children's Health, 100045, China.

**Keywords:** glial, heterotopia, intranasal, neoplasm

## Abstract

**Rationale:**

Nasal glial heterotopia is a rare type of neoplasm consisting of meningothelial and/or neuroglial elements.

**Patient concerns:**

A 2-month-old male was evaluated for treatment of a congenital mass in the right nasal cavity near the pharynx.

**Diagnoses:**

The patient was preoperatively diagnosed with a congenital intranasal neoplasm.

**Interventions:**

Nasal endoscopic resection of the nasopharyngeal mass was performed under general anesthesia. Histological examination of the resected tissue provided a diagnosis of intranasal glial heterotopia.

**Outcomes:**

The surgical outcome was good, with no surgical site infection. After 1 year of follow-up, the boy was asymptomatic with no recurrence.

**Lessons:**

Excision of a nasopharyngeal mass via nasal endoscopy resulted in no recurrence during 1 year of follow-up. Before any surgical treatment for suspected glial heterotopia, the mass should be differentiated clinically and radiologically from an encephalocele to prevent the risk of cerebrospinal fluid leakage and meningitis.

## 1 Introduction

Glial heterotopia is a rare congenital developmental disorder in which neuroglial tissue forms in extracranial sites, usually in the midline. As glial heterotopia commonly occurs in or around the nose, it is often referred to as nasal glial heterotopia (NGH).^[1]^ NGH is a rare neoplasm consisting of meningeal and/or glial components that was first described in 1852.^[2]^ The incidence of congenital nasal masses is reportedly 1 in 20,000 to 40,000 live births,^[3–7]^ and NGH accounts for approximately 5% of all congenital nasal masses.^[8]^ Congenital nasal masses are thought to develop due to faulty closure of the anterior neuropore. Most of these lesions arise from the lateral nasal wall and cause nasal obstruction.^[6]^ Herein, we report a case of a 2-month-old male with NGH and review similar cases reported in the literature.

## 2 Case report

**Figure 1:**
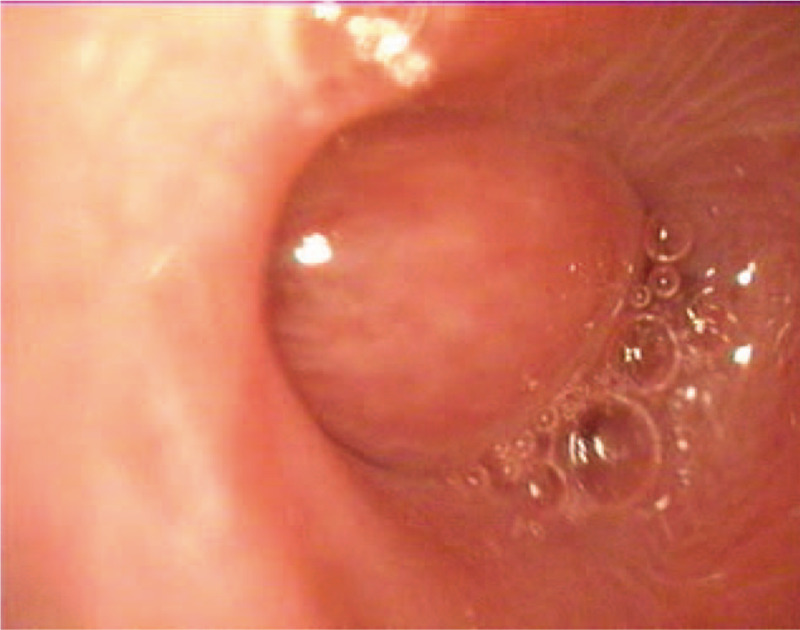
Preoperative bronchoscopy shows a round mass obstructing the right posterior nostril, with no pulsation and no obvious change in the size of the mass during breathing.

**Figure 2:**
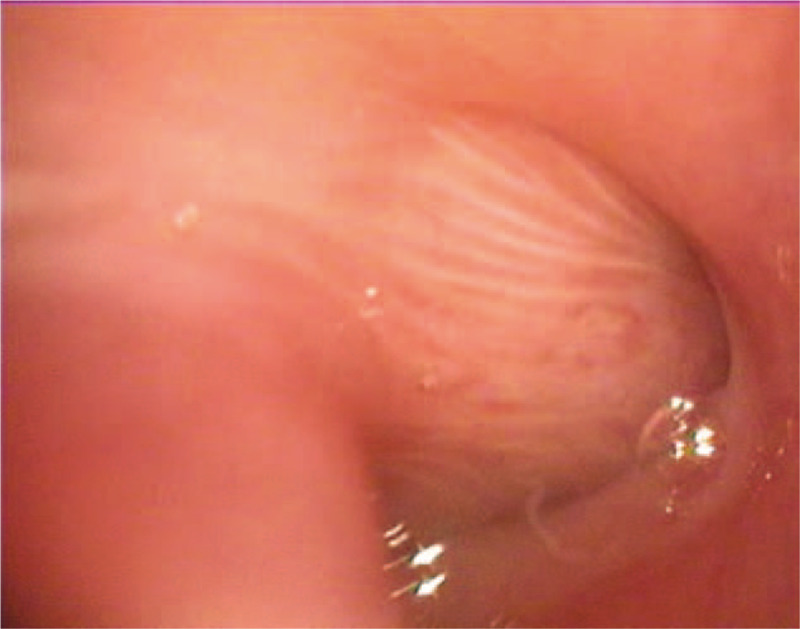
Preoperative bronchoscopy shows a large, broad-based mass in the left nasopharynx with a smooth surface and an upper margin on the posterior nasal plane.

**Figure 3:**
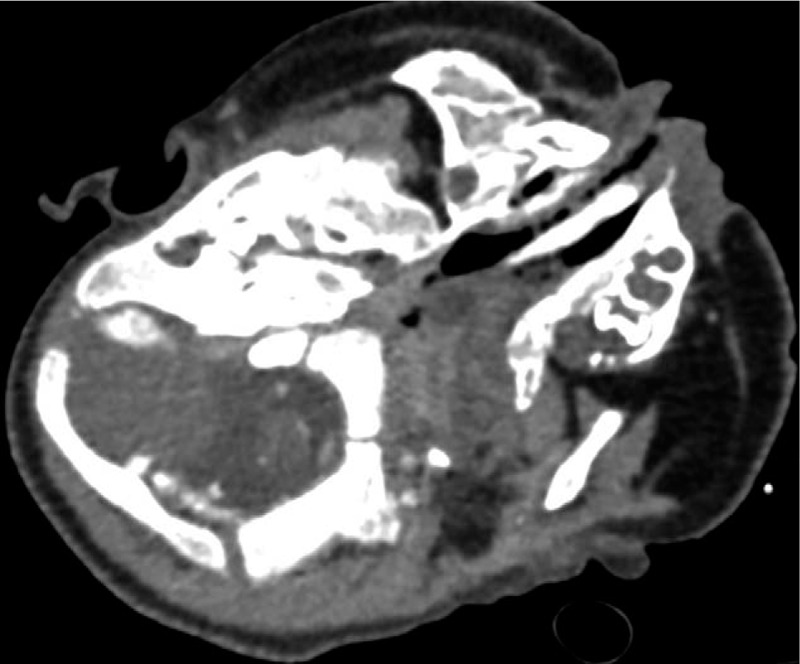
Nasopharyngeal contrast-enhanced computed tomography shows a soft tissue density mass in the left nasopharyngeal cavity and the posterior wall of the oropharyngeal cavity. The lesion is 3 × 1.9 × 1.9 cm, and is unevenly enhanced with an unclear boundary between the lesion and nasopharyngeal adipose tissue.

**Figure 4:**
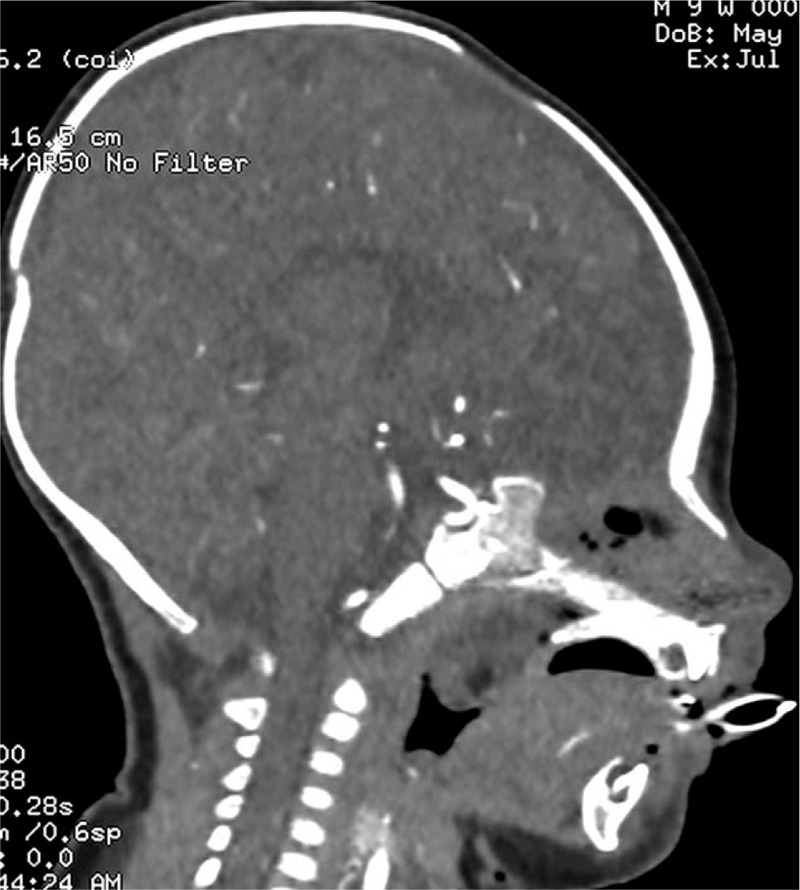
Nasopharyngeal contrast-enhanced computed tomography showing that the large wing of the left sphenoid bone is thinner and less continuous than normal; the inner plate of the left wing is not shown.

A 2-month-old male presented with nasal obstruction and shortness of breath since birth; he had not had a fever, had normal mental responses, and was passing normal stools and urine. On the 22nd day after birth, the nasal obstruction worsened, and the patient had difficulty breathing, a bluish face, and a decreased heart rate. The patient was given cardiopulmonary resuscitation and started on ventilation via tracheal intubation. On the 35th day after birth, fiberoptic bronchoscopy showed a 1.5 × 1.5 cm polypoid substance in the right nasal cavity near the pharynx (Fig. 1), and the bronchoscope could not easily pass through the left nasal cavity (Fig. 2). Bronchoscopic examination of the larynx revealed chondroid cartilage, and epiglottitis; the patient choked as the scope passed the glottis, and so a thorough epiglottis root exploration was not performed. In the main airway, there was obvious mucosal congestion necessitating infection control and other treatment. Nasopharyngeal contrast-enhanced computed tomography (CT) showed soft tissue density in the left nasopharyngeal cavity and the posterior wall of the oropharyngeal cavity (Figs. 3 and 4). The soft tissue mass was about 3 × 1.9 × 1.9 cm, and was unevenly enhanced. The boundary between the lesion and nasopharyngeal adipose tissue was not clear, and the bone of the left sphenoid flank was thinner than normal.

**Figure 5:**
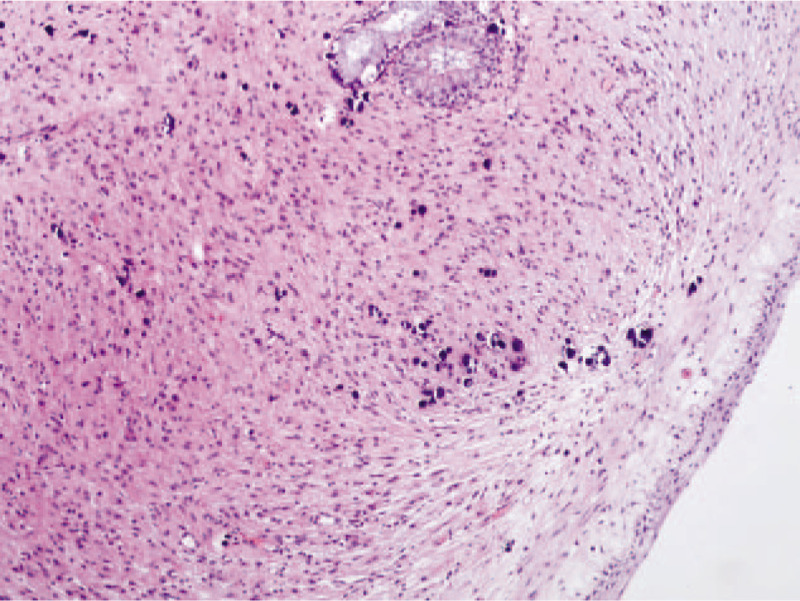
Histopathological examination shows a neuroglial heterotopia composed of glial cells and neuroglial fibers. Hematoxylin and eosin stain, ×100 magnification.

**Figure 6:**
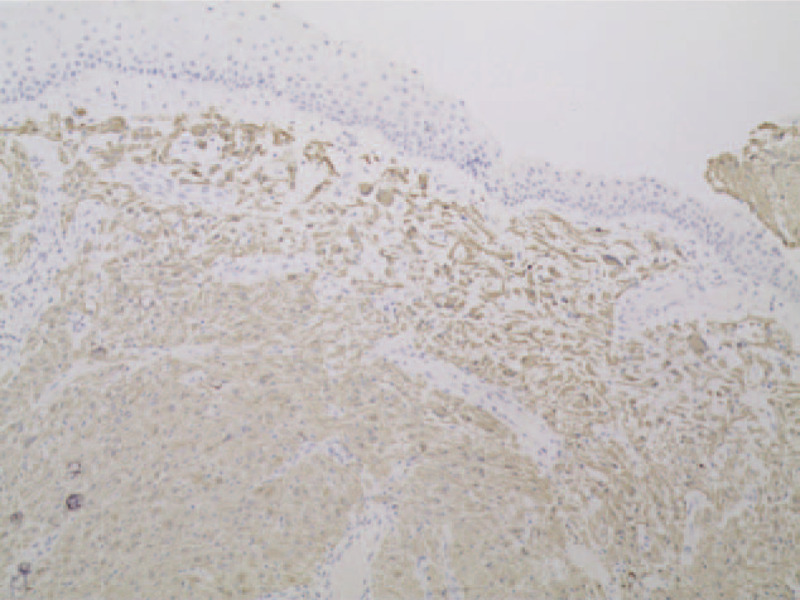
Immunohistochemical examination shows that the neuroglial tissue stained positive for glial fibrillary acidic protein. ×100 magnification.

**Figure 7:**
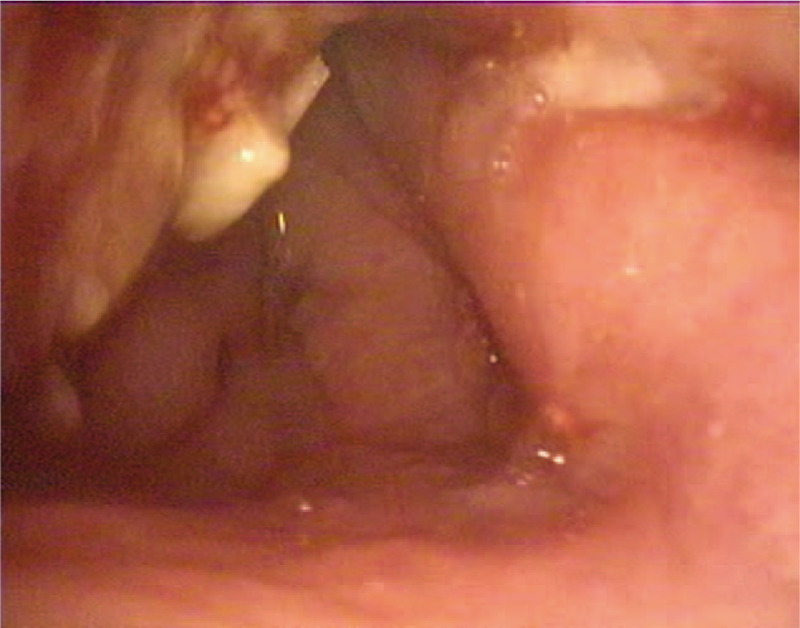
Bronchoscopy performed 1 week postoperatively showing the unobstructed nasopharynx with a few pseudomembranes on the lateral wall of the left nasopharynx.

A nasal endoscope was used to excise the nasopharyngeal mass under general anesthesia. Intraoperatively, it was found that the lobulated mass had a broad base that originated from the lateral wall of the left nasopharynx, with an upper boundary that reached the posterior nostril and a lower boundary that reached the upper polar plane of the tonsil; the mass was the same color as the mucosa. A multipoint puncture without liquid was performed, the tumor was resected with a low-temperature plasma knife, and the wounds were sutured. Pathological examination revealed that the mass was a glial heterotopia (left nasopharynx) (Fig. 5). The immunohistochemical results were positive for glial fibrillary acidic protein and microtubule-associated protein 2, and negative for NeuN (Fig. 6). The dyspnea was completely relieved after surgery. The nasopharyngeal patency was good at 1 week postoperatively (Fig. 7). There was no recurrence during 1 year of follow-up. The patient's parents provided written informed consent for the publication of the case details.

## 3 Discussion

A congenital midline nasal mass is a rare developmental abnormality. NGH can be thought of as an encephalocele that has lost its intracranial connections. Most reported cases of NGH are unilateral nasal or extranasal orbital, nasopharyngeal, middle ear, or scalp swellings.^[1]^ NGH is mainly located in or near the nasal cavity; 60% of cases are located outside the nose, 30% are located inside the nose, and 10% are located outside and inside the nose,^[9,10]^ and only 15% to 20% show intracranial communication.^[6,11]^ In our case, the mass was intranasal. Although glial heterotopias are benign lesions, they can cause clinical problems depending on their location. Intranasal lesions may be accompanied by nasal obstruction, epistaxis or nasal deformity. Intranasal glial heterotopias can cause nasal congestion and dyspnea, as seen in our case.

In 15% of patients with NGH, nerve growth hormone connects the lesion to the dura mater via a fibrous stalk,^[12]^ but there is no fluid to fill the space connecting the lesion to the subarachnoid space. In the current case, the lesion was identified at birth, and there was no fibrous stalk connecting the swelling with the intracranial space. Glial tissue can be confirmed by assessing the immune response to glial fibrillary acidic protein or S100 protein. Neurons are rare or absent in NGH, consistent with our observations in the present case. Although ependymal tissue is not always recognized in encephaloceles, its presence is more likely to lead to a diagnosis of encephalocele.^[6]^ On histological examination, it is usually impossible to distinguish NGH from encephalocele, as the two lesions can contain different proportions of neurons and glia. NGH must be distinguished from encephalocele, as the two lesions have similar embryological origins and can both manifest as intranasal masses. When the encephalocele is accompanied by meninges, it is termed a meningoencephalocele.

Nasal encephaloceles are a subtype that may be frontoethmoidal or basal.^[13]^ In frontoethmoidal encephalocele, brain tissue herniates through a defect of the frontal or ethmoidal bone into the soft tissues of the forehead, nose, and orbit, and is designated as nasofrontal, nasoethmoidal, or nasoorbital, respectively. The embryopathogenesis of nasal encephaloceles is controversial.^[14]^ Basal encephaloceles occur in the nasal cavity rather than as an external mass, and their developmental herniation is posterior to the cribriform plate. The incidence of related developmental abnormalities in patients with encephalocele varies from 0% to 40%.^[2]^ The most common site of encephalocele is occipital (75%), followed by the frontal lobe (25%). Nasal neuroglial heterotopia and encephalocele are very rare diseases that require multidisciplinary evaluation and management.^[15]^

The clinical manifestations of NGH are similar to those of congenital hemangioma (CH), and it is difficult to diagnose CH and NGH using prenatal ultrasonography. The blood flow velocity in Doppler examination is fast for CH and slow for NGH. On magnetic resonance imaging (MRI), the two lesions show high signal intensity on T2-weighted sequence, but the intensity of NGH is lower than that of CH.^[16]^ Although it may be difficult to distinguish NGH from CH, this has no direct effect on treatment, as both lesions require surgical treatment.^[16]^

CT and MRI are important in the diagnosis of NGH. CT is helpful in assessing bone defects, although CT may have different effects on young children whose bone structure is not yet fully formed.^[17]^ Bone defects in patients with developmental abnormalities may be associated with NGH, but may not be associated with intracranial tissue.^[17]^ MRI is superior to CT in providing the details of soft tissue, and it is more valuable in identifying an intracranial connection. As our patient had dyspnea, tracheal intubation and ventilator-assisted breathing were instigated and MRI was not performed; thus, CT was used to exclude intracranial extension.

Preoperative biopsy and resection are prohibited without preoperative imaging to determine the extent and location of the mass and exclude any connection with the central nervous system; this is to prevent complications such as cerebrospinal fluid (CSF) leakage, meningitis or encephalocele.^[8]^ Thus, the present patient was not biopsied preoperatively.

The preferred treatment for NGH is complete surgical excision. NGH grows slowly and benignly, with no possibility of malignant transformation.^[18]^ However, early surgical treatment is advocated because the growth of gliosis may lead to deformity and erosion of the facial bones.^[18]^ Furthermore, early surgical treatment may avoid serious complications such as meningitis, brain abscess, nasal septum and nasal bone deformation.^[19]^ The specific surgical method should be determined in accordance with the location and size of the mass. Transnasal endoscopic surgery is recommended for intranasal glial heterotopia. Due to advances in endoscopic equipment and technology, intranasal glial heterotopia can now be properly exposed and completely removed.^[6]^ For most intranasal and mixed NGH, endoscopic sinus surgery is feasible, with no increase in operation time, residual disease or complications.^[15]^ In addition, inadequate primary resection results in a recurrence rate of 4% to 10%.^[20]^ Our patient had no recurrence during 1 year of follow-up. Thorough preoperative imaging is necessary before glioma resection.

## 4 Conclusion

We report a rare case of NGH, which is a congenital developmental anomaly rarely reported in the literature. Before any form of surgical treatment for suspected glial heterotopia, the lesion should be differentiated clinically and radiologically from encephalocele to prevent the risk of CSF leakage and meningitis. Clinical examination supplemented by imaging investigations (such as MRI and CT) helps to achieve early diagnosis and timely surgical consultation. To prevent the risk of CSF leakage, the possibility of intracranial connections must be considered when planning surgery for congenital midline masses. Conservative surgical excision remains the accepted modality of treatment due to the rare recurrence and nonmalignant potential of NGH.

## Acknowledgments

We thank Kelly Zammit, BVSc, from Liwen Bianji, Edanz Editing China, for editing the English text of a draft of this manuscript.

## Author contributions

**Investigation:** Wei Zhang, Lixing Tang.

**Resources:** Wentong Ge, Lixing Tang.

**Supervision:** Xin Ni.

**Writing – original draft:** Wei Zhang.

**Writing – review and editing:** Pengpeng Wang.
